# In Silico Analysis of the L-2-Hydroxyglutarate Dehydrogenase Gene Mutations and Their Biological Impact on Disease Etiology

**DOI:** 10.3390/genes13040698

**Published:** 2022-04-15

**Authors:** Muhammad Muzammal, Alessandro Di Cerbo, Eman M. Almusalami, Arshad Farid, Muzammil Ahmad Khan, Shakira Ghazanfar, Mohammed Al Mohaini, Abdulkhaliq J. Alsalman, Yousef N. Alhashem, Maitham A. Al Hawaj, Abdulmonem A. Alsaleh

**Affiliations:** 1 Gomal Centre of Biochemistry and Biotechnology, Gomal University, Dera Ismail Khan 29050, Pakistan; mustafamuzammal@gu.edu.pk (M.M.); muzammilgandapur1983@gmail.com (M.A.K.); 2 School of Biosciences and Veterinary Medicine, University of Camerino, 62024 Matelica, Italy; 3 King’s College London, Strand, London WC2R 2LS, UK; eman.al_musalami@kcl.ac.uk; 4 Department of Human Genetics, Precision Medicine Program, Sidra Medicine, Doha 26999, Qatar; 5 National Institute for Genomics Advanced Biotechnology, National Agricultural Research Centre, Park Road, Islamabad 45500, Pakistan; shakira_akmal@parc.gov.pk; 6 Basic Sciences Department, College of Applied Medical Sciences, King Saud bin Abdulaziz University for Health Sciences, Alahsa 31982, Saudi Arabia; mohainim@ksau-hs.edu.sa; 7 King Abdullah International Medical Research Center, Alahsa 31982, Saudi Arabia; 8 Department of Clinical Pharmacy, Faculty of Pharmacy, Northern Border University, Rafha 91911, Saudi Arabia; kaliqs@gmail.com; 9 Clinical Laboratory Sciences Department, Mohammed Al-Mana College for Medical Sciences, Dammam 34222, Saudi Arabia; yousefa@machs.edu.sa (Y.N.A.); a.alsaleh@machs.edu.sa (A.A.A.); 10 Department of Pharmacy Practice, College of Clinical Pharmacy, King Faisal University, Al-Ahsa 31982, Saudi Arabia; hawaj@kfu.edu.sa

**Keywords:** L2HGDH, metabolism, in silico analysis, modeling and docking

## Abstract

The L-2-hydroxyglutarate dehydrogenase (L2HGDH) gene encodes an important mitochondrial enzyme. However, its altered activity results in excessive levels of L-2-hydroxyglutarate, which results in diverse psychiatric features of intellectual disability. In the current study, we executed an in-silico analysis of all reported L2HGDH missense and nonsense variants in order to investigate their biological significance. Among the superimposed 3D models, the highest similarity index for a wild-type structure was shown by the mutant Glu336Lys (87.26%), while the lowest similarity index value was shown by Arg70* (10.00%). Three large active site pockets were determined using protein active site prediction, in which the 2nd largest pocket was shown to encompass the substrate L-2-hydroxyglutarate (L2HG) binding residues, i.e., 89Gln, 195Tyr, 402Ala, 403Gly and 404Val. Moreover, interactions of wild-type and mutant L2HGDH variants with the close functional interactor D2HGDH protein resulted in alterations in the position, number and nature of networking residues. We observed that the binding of L2HG with the L2HGDH enzyme is affected by the nature of the amino acid substitution, as well as the number and nature of bonds between the substrate and protein molecule, which are able to affect its biological activity.

## 1. Introduction

L-2-hydroxyglutaric aciduria (L2HGA, OMIM #236792) is a very rare genetic condition of the metabolism. It occurs due to null or reduced action of L-2-hydroxyglutarate dehydrogenase (L2HGDH), a mitochondrial enzyme that catalyzes the oxidation of L-2-hydroxyglutaric acid into α-ketoglutarate [1,2,3]. L2HGDH is encoded by the L2HGDH gene and is an autosomal recessive condition that occurs due to an increased level of L-2-hydroxyglutaric acid in the urine, plasma and cerebrospinal fluid (CSF) [1].

The phenotypical traits of the individuals affected with L2HGA are variable, and may comprise macrocephaly, cerebellar ataxia, developmental delay spasticity, variable range of intellectual disability, epilepsy, communication problems and difficulties in speech [4,5,6,7,8].

The inception of L2HGA is also different, e.g., it might be either congenital with severe seizures and intellectual disability or might occur in middle age with mild features, which is an issue that may result in a delayed diagnosis of L2HGA [9,10,11]. Many studies have reported that elevated levels of L-2-hydroxyglutaric acid in the brain, probably linked with higher levels of lysine in the blood, may cause brain tumors [3,12,13].

Early anamnesis and treatment may decelerate the development of the disorder. In this sense, due to the uncertainty regarding the different phenotypes of metabolism disorders, a complete neurological checkup must be a component of regular pediatric appointments [1,2]. Radiological tests e.g., MRI and CT scans, may be useful in identifying patients who are supposed to have inborn metabolism alterations [8,14].

Hijikata and his coworkers documented that a major structural location of autosomal recessive mutations is the buried region of a protein, while the mutations cause the destabilization of the protein structure [15]. A Bayesian hierarchical method was also validated to study the disease causality of missense mutations due to the impossibility to predict the structural and functional changes of the protein following an amino acid substitution [16]. Agrahari et al. reported that missense mutations effect the stability as well as dynamics of the protein and results in disease phenotypes [17,18].

So far, the Human Gene Mutation Database (HGMD) has registered 52 missense or nonsense variants in the L2HGDH gene, which are reported to disturb the activity of its enzyme and have been documented in different ethnicities around the world. Previously, in Pakistan just 2 mutations, c.C751T (p.Arg251*) and c.G178A (p.Gly60Arg), have been described [19,20].

The current study intended to evaluate the biological effects of all missense or nonsense mutations reported in the L2HGDH gene via protein modeling and docking.

## 2. Materials and Methods

The data for all L2HGDH described variants were retrieved from the HGMD list [21], while the wild-type protein sequence was retrieved from Ensembl genome browser https://www.uniprot.org/uniprot/Q9H9P8, (accessed on 1 October 2021) [22]. To predict the structural model of wild-type and all L2HGDH mutants, 3D models were designed using the RaptorX tool and cross-checked using the I-TASSER tool [23]. Among the designed models, those with the highest confidence scores (CS) were chosen for the study. The 3D models were visualized using the Pymol and Chimera tools [24]. To examine the alterations produced by mutations, 3D models of wild-type and mutant L2HGDH proteins were superimposed individually using Chimera. Nonetheless, active sites and binding pockets of wild-type L2HGDH enzyme were predicted using the CASTp tool [25]. We used default settings for all tools used in the current in silico study.

Protein–protein interactions for wild-type and all mutant L2HGDH variants with their close functional interactor D2HGDH protein were assessed using Cluspro [26]. The close functional interactor of L2HGDH, D2HGDH, was predicted using the String v9.1 database (https://string-db.org, accessed on 1 October 2021) [27]. Likewise, protein–substrate docking of wild-type and mutant L2HGDH variants with the substrate 2-hydroxyglutarate (L2HG) was performed using Autodock Vina in addition to MGL tools [28]. The protein–substrate docked structures were visualized using the offline tool Discovery Studio 2020 Client [29]. Protein–substrate simulations were performed using the iMODs tool available at www.imods.iqfr.csic.es (accessed on 10 January 2022); iMODS works on the normal mode analysis (NMA) principal. The results of the simulation were calculated in the form of an elastic network, covariance map, eigenvalues and the mobility of the complex using default basic settings.

## 3. Results

### 3.1. Structural Analysis

All predicted 3D structures of L2HGDH mutations (Supplementary Figure S1) were superimposed with a wild-type L2HGDH model (Supplementary Figure S2). The physical evaluation of these representations showed notable variances in the structure, which were noted in the form of the percentage identities.

Amongst the mutated protein models, the maximum resemblance index of wild-type L2HGDH protein with mutant protein was presented by Glu336Lys (87.26%), whereas the lowermost resemblance index was presented by mutant Arg70*, which was 10.00% (Supplementary Figure S2). A similarity index with the wild-type protein’s 3D structure was assessed using the Chimera tool.

Table 1 summarizes the percentage identity values of all mutant models with the wild-type L2HGDH protein.

### 3.2. Active Site Predication

The top three largest active sites pockets were predicted in the wild-type L2HGDH protein using the CASTp tool. Among three largest predicted active site pockets, wild-type L2HGDH protein amino acids that were involved in binding with the substrate molecule L2HG were present in the second largest active site pocket. Wild-type protein amino acids interacting with the substrate were 89Gln, 195Tyr, 402Ala, 403Gly and 404Val. The complete explanation of wild-type L2HGDH protein residues and their positions, existing in these three largest active site pockets, are summarized in Table 2.

**Figure 1:**
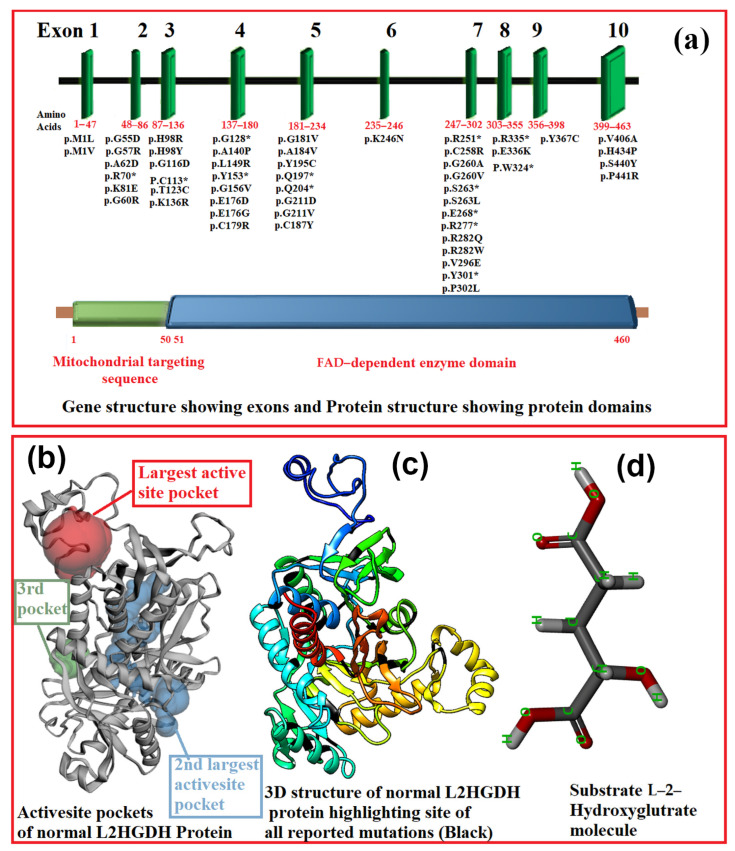
(**a**) L2HGDH gene and the positions of the mutations of respective exon and L2HGDH protein structures. (**b**) A 3D model of wild-type L2HGDH protein and its active site pockets. (**c**) The 3D structure of normal L2GHDH protein and the sites of all reported mutations in black. (**d**) L2HGDH substrate of L2HG.

The active site pockets of the L2HGDH protein are illustrated in Figure 1.

### 3.3. Protein–Protein Interaction

Protein–protein interactions were assessed between all mutant and wild-type L2HGDH proteins with their close functional interactor D2HGDH protein and notable alterations were observed in the interrelating sites of wild-type and all mutant L2HGDH proteins. The interactions showed that the wild-type L2HGDH protein interacted with the interactor D2HGDH protein through fourteen different amino acids, i.e., Asp194, Ser31, Arg16, Ser23, Gly13, Arg14, Thr346, Gln125, Lys292, Glu291, Arg33, Gly32, Arg35 and Cys27, via seventeen interactive hydrogen bonds. Nonetheless, among all L2HGDH mutant proteins, the greatest interaction was shown by Arg335*, which interacted with the D2HGDH protein through twenty-seven bonds (twenty-four hydrogen and three salt bridges) and twelve different amino acids. The lowest levels of interaction were shown by Ala406Val and Pro302Leu proteins, wherein both the mutants (Ala406Val and Pro302Leu protein) interacted with D2HGDH through two hydrogen bonds involving two different amino acids (each). Illustrative images of all protein–protein interactions between wild-type and mutant L2HGDH proteins with their close functional interactor D2HGDH are presented in Supplementary Figure S3A–D.

### 3.4. Protein–Substrate Docking

Protein–substrate docking was performed to better understand the interaction of L2HG with the wild-type and L2HDGH mutant proteins. Wild-type L2HGDH docked with L2HG through five hydrogen bonds via five amino acids, i.e., Gln89, Tyr195, Ala402, Gly403 and Val404. The highest levels of protein–substrate docking was present in Gly211Asp and Arg282Gln variants, wherein the mutant Gly211Asp protein docked with L2HG via seven bonds (five hydrogens, two carbon–hydrogens) and five different amino acids, while nine residues characterized by Van der Waals interactions with the substrate molecule were also observed.

Mutant Arg282Gln interacted with L2HG through seven bonds (one hydrogen and one carbon–hydrogen) via four different residues, while Van der Waals interactions were also noted for six different amino acids.

The lowest level of protein–substrate docking was noted in the Tyr301* variant. No protein–substrate docking was present between the mutant Tyr301* and substrate molecule, since it resulted in a short-truncated protein; nevertheless, nine different amino acids interacting through Van der Waals forces were observed around the substrate molecule.

Every interacting amino acid of mutant L2HGDH proteins with L2HG was changed in comparison to the wild-type. Here, 2D illustrations of protein–substrate interactions amongst wild-type and mutant L2HGDH proteins by L2HG are presented in Supplementary Figure S4A–C.

### 3.5. Molecular Simulation

**Figure 2:**
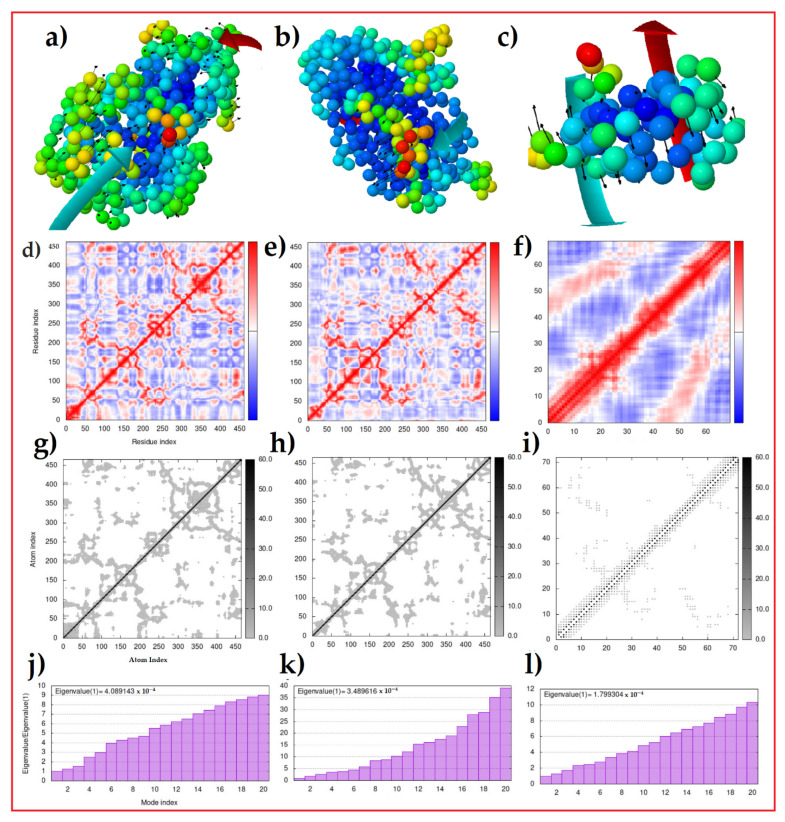
(**a**–**c**) The complexes of wild-type, mutant Glu336Lys and Arg70* variants with substrate L2HG. (**d**–**f**) The covariance matrixes of wild-type, mutant Glu336Lys and Arg70* variants with substrate L2HG. (**g**–**i**) The results of the elastic network models of wild-type, mutant Glu336Lys and Arg70* variants with substrate L2HG. (**j**–**l**) The eigenvalues of wild-type, mutant Glu336Lys and Arg70* variants with substrate L2HG.

We calculated the index values of wild-type and mutant Glu336Lys variants, having the highest similarity index values, and mutant Arg70* variants, having the lowest similarity index values, with the wild-type L2HGDH protein and substrates of L2HG individually (Figure 2). Figure 2a,c shows a clear difference in the orientations of residues between the wild-type-L2HG complex and the mutant Arg70*-L2HG complex, while the residues in the wild-type and Glu336Lys variants are much similar. In the covariance matrix, the map indicates coupling between pairs of residues, i.e., whether they experienced correlated (red), uncorrelated (white) or anti-correlated (blue) motion. In Figure 2d,e, a difference can be seen between the correlating residues of wild-type-L2HG and Glu336Lys--2-HG complexes on the map, but this difference was much more obvious in the correlated residues of the Arg70*-L2HG complex.

We also calculated the elastic network, which defines the pairs of atoms that are connected by springs. Each dot in the graph represents one spring between the corresponding pair of atoms. Dots are colored according to their stiffness, whereby the darker grays indicate stiffer springs and vice versa. The stiffness levels of the complexes are shown in Figure 2g–i. In Figure 2g, the stiffness of the wild-type substrate complex is much higher with respect to mutant Glu336Lys-L2HG (Figure 2h) and Arg70*-L2HG complexes (Figure 2i). These results were also verified through the eigenvalues. The eigenvalue associated with each normal mode represents the motion stiffness. Its value is directly related to the energy required to deform the structure. The lower the eigenvalue, the easier the deformation. In Figure 2j, we show the eigenvalue of the wild-type substrate complex, which was 4.089, the mutant Glu336Lys-substrate L2HG complex, which was 3.489, and the Arg70*- substrate L2HG complex, which was 1.799. These results show that complex of wild-type substrate L2HG was much more stable as compared to that of the mutant Glu336Lys-L2HG, while the least stable complex was that of Arg70*-L2HG. These results confirm the quality of those achieved via molecular modeling and docking.

## 4. Discussion

L2HGDH (NM#024884.3) is a protein-coding gene and is positioned on the chromosome number 14. It comprises ten exons with an estimated size of 75 Kb, which transcribe five transcripts for L2HGDH proteins [1]. L2HGDH is a mitochondrial protein that is implicated in the metabolism of butanoate and glutamate, along with glutamine metabolism paths.

The protein is composed of 463 amino acids with two domains, one of which is a mitochondrial targeting sequence (aa 1–50), while the other one is an FAD-dependent domain.

L2HGDH is expressed in a variety of tissues such as the brain, muscles and testes. An important purpose of the enzyme is to be a catalyst of the oxidation reaction of L2HG into α2-ketoglutarate [37]. The functional deficiency of the activity of this enzyme increases the concentration of L-2-hydroxyglutaric acid up to a lethal level in plasma, cerebrospinal fluid and urine. A mutation in the L2HGDH gene is accountable for a rare neurodegenerative condition of the metabolism termed L2HGDA. The distinctive physical features linked to L2HGDA consist of psychomotor anomalies, intellectual disability, leukodystrophy, macrocephaly, tremors, epilepsy and abnormal posture coupled with cerebellar degeneration [9]. The resultant phenotypes can also be observed in knockout mice with L2HGDH. So far, only two mutations, i.e., c.1003C > T p.(Arg335*) [16] and c.178G > A p.(Gly60Arg) [19], have been described in Pakistani families. The first mutation reported in the Pakistani population was the nonsense mutation p.Arg335*, which showed a similarity index of 75.82% with normal L2HGDH protein, while p.Gly60Arg was the second missense mutation reported in Pakistan, showing a similarity index of 79.05% with normal L2HGDH protein. Although the prevalence of disabilities in Pakistan is much higher, further studies are needed to explore in detail the rates of these types of metabolic disorders.

Penderis et al. (2007) documented a dog model of aciduria in bull terriers, which were outbred. All animals displayed excessive urinary secretion of L2HG, whereas twelve dogs subjected to MRI imaging exhibited symmetric sections of hyperintensity similar to that observed in L2HGDA patients [39].

Likewise, Ma et al. (2017) established a null mice model for a variety of physical features, i.e., excessive L2HG concentrations in various tissues, particularly in the brain and testes. Moreover, the model exhibited white matter weakening and massive gliosis, microglia-mediated neuroinflammation coupled with the development of oligodendrocyte precursor cells. Furthermore, L2HGDH defects in subsequent phases cause hippocampal neurogenesis and late-inception neurodegeneration [40].

The precise frequency of L2HGDA (OMIM#236792) has still not been identified, and roughly one hundred and forty-four incidents have occurred so far, with one hundred mutations of L2HGDH having been recorded in various ethnic groups worldwide [3,8,13]. The HGMD databank has registered fifty-two missense or nonsense mutations, which are implicated in the reduced action of the L2HGDH enzyme. In this study, we attempted to examine the physical effects of all stated mutations in L2HGDH and tried to connect it with its enzymatic action.

Each L2HGDH mutation demonstrated variable outcomes in terms of the 3D shapes and attachments to the respective substrate, the L2HG.

Normal L2HGDH protein attached to the substrate molecule through five hydrogen bonds, but we observed extra bonding in all mutant proteins, i.e., unfavorable bonds, carbon–hydrogen bonds and Van der Waal forces were produced around the protein–substrate complexes of mutant and substrate molecules. Hydrogen bond interactions are comparatively fragile interactions; nonetheless, they are vital for biological macro-molecule formation, i.e., for DNA and proteins. Wendler et al. (2010) documented that hydrogen bonds are comparatively less strong than covalent bonds [41]. Hydrogen bonds have energy levels of around 1–3 kcal mol^−1^, in contrast with the roughly 100 kcal mol^−1^ of energy for covalent bonds formed between carbon and hydrogen.

The bond distance for hydrogen bonds is slightly longer compared to covalent bonds; when measured from the hydrogen atom, the bond distances range between 1.5 and 2.6 Å, while the bond distances range between 2.4 and 3.5 Å among non-hydrogen atoms when held together through a hydrogen bond. A strong hydrogen bond is inclined to form an almost straight bond, i.e., a bond established amongst a hydrogen bond donor and a hydrogen bond acceptor through a hydrogen atom lies beside a straight line [41,42,43].

In the present study, we observed hydrogen bond interactions between wild-type L2HGDH proteins with their substrate, but in the case of the L2HGDH mutant protein, we observed the presence of covalent bonds (carbon–hydrogen bonding) along with some other non-specific bonds. Furthermore, all mutant proteins had Van der Waal interactions that may affect the activity of the L2HGD enzyme.

Although energies related with Van der Waals forces are small, normal interactions provide 0.5–1.0 kcal mol^−1^/atom pair, although when two big molecules interact, a huge number of atoms come in contact by means of Van der Waals interactions, producing a net effect, which is summated over numerous atom pairs and can be significant.

Roth et al. documented that Van der Waals forces are involved in the contact of proteins with several different molecules directly or indirectly with their exteriors, but due to the intricacy of the protein structure, the degree of these consequences is usually considered based on their (protein) perfect structure, i.e., spheroids or spheres. The shape of the molecular has also been shown to affect the magnitude of the interactions. Regarding the interactions occurring in the spherical protein structure, an initial coarseness of the molecular surfaces led to lower standard interaction forces for protein–protein and protein-surface instances relative to interactions in which the protein was assessed as a sphere. The results found by Roth et al. indicated that a form of steric stabilization might be vital in protein interactions [44].

In normal protein substrates, interactive hydrogen bonds were present, which were weaker as compared to the carbon–hydrogen bonds that were present in most of the mutant proteins. Moreover, many Van der Waals forces were also present in mutant protein–substrate interactions, which possibly interfered with protein–substrate interactions and affected the enzymatic activity of the L2HGDH enzyme, resulting in the disease phenotype.

## 5. Conclusions

The current in silico study was carried out to verify the effects of all reported missense and nonsense L2HGDH mutations on interactions of L2HG with its protein by means of different modeling and docking techniques. From the results, we noticed that the type of amino acid replacement, the sum of the bonds and the presence of Van der Waal forces modify the binding and dissociation of the L-2-hydroxyglutaric acid molecule with the L2HGDH enzyme, thereby altering its action. Additionally, we noticed that the severity of damage to L2HGDH varies with the type of mutation. No similar study on L2HGDH gene has been documented so far, meaning this is the first in silico study on all reported L2HGDH mutations, which can provide a better understanding of the topic for researchers and scientists in the field of genetics and metabolic disorders.

## Figures and Tables

**Table 1:** Summary of the nature of amino acid substitution and the type and number of bonds between the mutant L2HGDH and substrate molecule.

Missense Mutation and Their Similarity Index with Wild Type Protein 3D Structure		Number of Bonds	H Bond	C-H Bond	Unfavorable Bond	Van Der Waals Forces	Wild-Type Amino Acid	Substituted Amino Acid	References
Wild type		5	5	-	-	-	-	-	-
Missense Variants									
Met1Leu	81.64%	14	6	-	-	8	Nonpolar/Neutral	Nonpolar/Neutral	[3]
Met1Val	80.78%	13	3	-	-	10	Nonpolar/Neutral	Nonpolar/Neutral	[3]
Ala62Asp	80.13%	10	3	-	1	6	Nonpolar/Neutral	Acidic polar/Negative	[30]
Ala140Pro	79.27%	12	4	-	1	7	Nonpolar/Neutral	Nonpolar/Neutral	[20]
Ala184Val	82.94%	15	3	-	1	11	Nonpolar/Neutral	Nonpolar/Neutral	[3]
Ala406Val	78.83%	11	2	-	-	9	Nonpolar/Neutral	Nonpolar/Neutral	[3]
Cys179Arg	80.78%	13	2	-	1	10	Nonpolar/Neutral	Basic polar/Positive	[3]
Cys258Arg	84.45%	12	3	1	1	7	Nonpolar/Neutral	Basic polar/Positive	[31]
Glu176Asp	84.67%	13	4	1	1	7	Acidic polar/Negative	Acidic polar/Negative	[3]
Glu176Gly	80.13%	15	4	1	-	10	Acidic polar/Negative	Nonpolar/Neutral	[32]
Glu336Lys	87.26%	16	2	2	-	12	Acidic polar/Negative	Basic polar/Positive	[3]
Gly55Asp	81.21%	11	3	-	-	8	Nonpolar/Neutral	Acidic polar/Negative	[13]
Gly57Arg	81.64%	15	4	-	-	11	Nonpolar/Neutral	Basic polar/Positive	[33]
Gly116Asp	79.05%	13	6	-	-	7	Nonpolar/Neutral	Acidic polar/Negative	[3]
Gly156Val	80.13%	13	6	-	-	7	Nonpolar/Neutral	Nonpolar/Neutral	[20]
Gly181Val	80.13%	13	2	1	2	8	Nonpolar/Neutral	Nonpolar/Neutral	[34]
Gly211Asp	80.56%	16	5	2	-	9	Nonpolar/Neutral	Acidic polar/Negative	[3]
Gly211Val	82.94%	14	3	1	1	9	Nonpolar/Neutral	Nonpolar/Neutral	[22]
Gly260Ala	82.51%	9	3	-	1	5	Nonpolar/Neutral	Nonpolar/Neutral	[3]
Gly260Val	79.70%	14	3	-	-	11	Nonpolar/Neutral	Nonpolar/Neutral	[3]
His98Arg	80.13%	15	5	-	1	9	Basic polar/Positive, 10%, Neutral, 90%	Basic polar/Positive	[33]
His98Tyr	80.13%	14	4	-	1	9	Basic polar/Positive, 10%, Neutral, 90%	Polar/Neutral	[13]
His434Pro	79.05%	14	5	1	-	8	Basic polar/Positive, 10%Neutral, 90%	Nonpolar /Neutral	[33]
Lys81Glu	80.99%	10	1	1	-	8	Basic polar/Positive	Acidic polar	[32]
Lys246Asn	80.13%	11	2	-	-	9	Basic polar/Positive	Polar/Neutral	[3]
Leu149Arg	80.13%	14	3	1	-	10	Nonpolar/Neutral	Basic polar/Positive	[20]
Pro302Leu	81.86%	14	3	-	1	10	Nonpolar/Neutral	Nonpolar/Neutral	[13]
Arg282Gln	80.13%	13	6	1	-	6	Basic polar/Positive	Polar/Neutral	[35]
Arg282Trp	80.13%	13	5	1	-	7	Basic polar/Positive	Nonpolar/Neutral	[3]
Ser263Leu	80.13%	10	2	-	-	8	Polar/Neutral	Nonpolar/Neutral	[20]
Ser440Tyr	78.19%	16	4	-	2	10	Polar/Neutral	Polar/Neutral	[20]
Val296Glu	78.62%	12	2	-	-	10	Nonpolar/Neutral	Acidic polar/Negative	[8]
Tyr195Cys	79.91%	12	2	1		9	Polar/Neutral	Nonpolar/Neutral	[20]
Tyr367Cys	80.35%	11	5	-	-	6	Polar/Neutral	Nonpolar/Neutral	[3]
Cys187Tyr	57.02%	13	2	1	1	9	Nonpolar/Neutral	Polar/Neutral	-
Gly60Arg	79.05%	15	1	1	2	12	Nonpolar/Neutral	Basic polar/Positive	[19]
Thr123Cys	80.35%	14	3	1	-	10	Polar/Neutral	Nonpolar/Neutral	[9]
Pro441Arg	81.86%	14	3	2	-	9	Nonpolar/Neutral	Basic polar/Positive	[3]
Lys136Arg	80.78%	13	4	-	-	9	Basic polar/Positive	Basic polar/Positive	[36]
Nonsense Variants									
Glu268*	63.43%	10	1	1	1	7	-	-	[20]
Gly128*	37.50%	14	2	1	1	10	-	-	[3]
Gln197*	51.27%	8	3	1	1	3	-	-	[33]
Gln204*	73.53%	9	2	-	-	7	-	-	[3]
Arg70*	10.00%	10	3	-	-	7	-	-	[33]
Arg251*	74.90%	10	4	-	-	6	-	-	[20]
Arg277*	76.90%	9	3	-	1	5	-	-	[3]
Arg335*	75.82%	13	5	-	-	8	-	-	[13]
Ser263*	74.90%	14	4	-	-	10	-	-	[37]
Tyr153*	32.03%	12	3	-	1	8	-	-	[13]
Tyr301*	65.78%	11	-	-	-	11	-	-	[3]
Cys113*	39.82%	10	4	-	1	5	-	-	-
Trp324*	76.23%	9	2	-	-	7	-	-	[38]

**Table 2:** CASTp results showing active site pockets in L2HGDH protein.

Size of Pocket	Residues and Their Number in Active Site Pockets
1st	5Leu, 6Arg, 7Tyr, 8leu, 16Arg, 19Phe, 22GLy, 23Ser, 24Pro, 26Ala, 42Arg, 44Ala, 45Ser, 83Lys, 207Gln, 210Gly, 211Gly, 212Ser, 213val, 214Leu, 215Thr, 216Asn, 217Phe
2nd	54Val, 55Gly, 57Gly, 58Ile, 59val, 60Gly, 79Leu, 80Glu, 81Lys, 82Glu, 84Asp, 85Leu, 87Val, 88His, 89Gln*, 91Gly, 92His, 93Asn, 95Gly, 96Val, 102Tyr, 103Tyr, 104Lys, 107Ser, 148Arg, 151Ala, 152Leu, 155Lys, 195Tyr*, 217Phe, 218Glu, 219Val, 220Lys, 258Cys, 259Ala, 260Gly, 261Leu, 262Tyr, 263Ser, 265Arg, 266Ile, 269Leu, 280Pro, 281Phe, 282Arg, 283Gly, 285Tyr, 301Tyr, 304Pro, 305Asp, 306Ser, 309Pro, 310Phe, 314His, 326Gly, 328Asn, 329Ala, 334Lys, 367Tyr, 402Ala*, 403Gly*, 404Val*, 405Arg, 406Ala, 407Gln, 415Leu, 416Val, 417Glu, 418Asp, 419Phe, 435Val, 436Arg, 437Asn, 438Ala, 439Pro, 440Ser, 441Pro, 442Ala, 434Ala
3rd	90Thr, 91Gly, 94Ser, 95Gly, Ser130, 131Tyr, 132Lys, 192Ile, 193Val, 194Asp, 195Tyr, 197Gln, 318Arg, 319Met, 324Trp

* Active site residues were present in 2nd largest pocket.

## Data Availability

The computational data are stored in the password-protected personal computers of M.M. and M.A.K., which are available upon request.

## Supplementary Materials

The following supporting information can be downloaded at: https://www.mdpi.com/article/10.3390/genes13040698/s1. Figure S1: 3D models of all reported nonsense and missense mutant L2HGDH proteins, Figure S2: Superimposed 3D models of all missense mutant L2HGDH proteins with wild-type L2HGDH protein. (b) Superimposed 3D models of all reported nonsense mutant L2HGDH proteins with wild-type L2HGDH protein, Figure S3: (A–D) Protein–protein interactions of all reported L2HGDH protein mutations with its close interactor D2HGDH protein, Figure S4: (A–C) Protein–substrate docking of all reported protein mutations and normal L2HGDH protein with its substrate L-2-HG.
