# Supplementary figures and images for: In Silico Analysis of the L-2-Hydroxyglutarate Dehydrogenase Gene Mutations and Their Biological Impact on Disease Etiology
Source: Genes (Basel). 2022 Apr 15;13(4):698. doi: 10.3390/genes13040698 (PMC9028441; doi:10.3390/genes13040698)

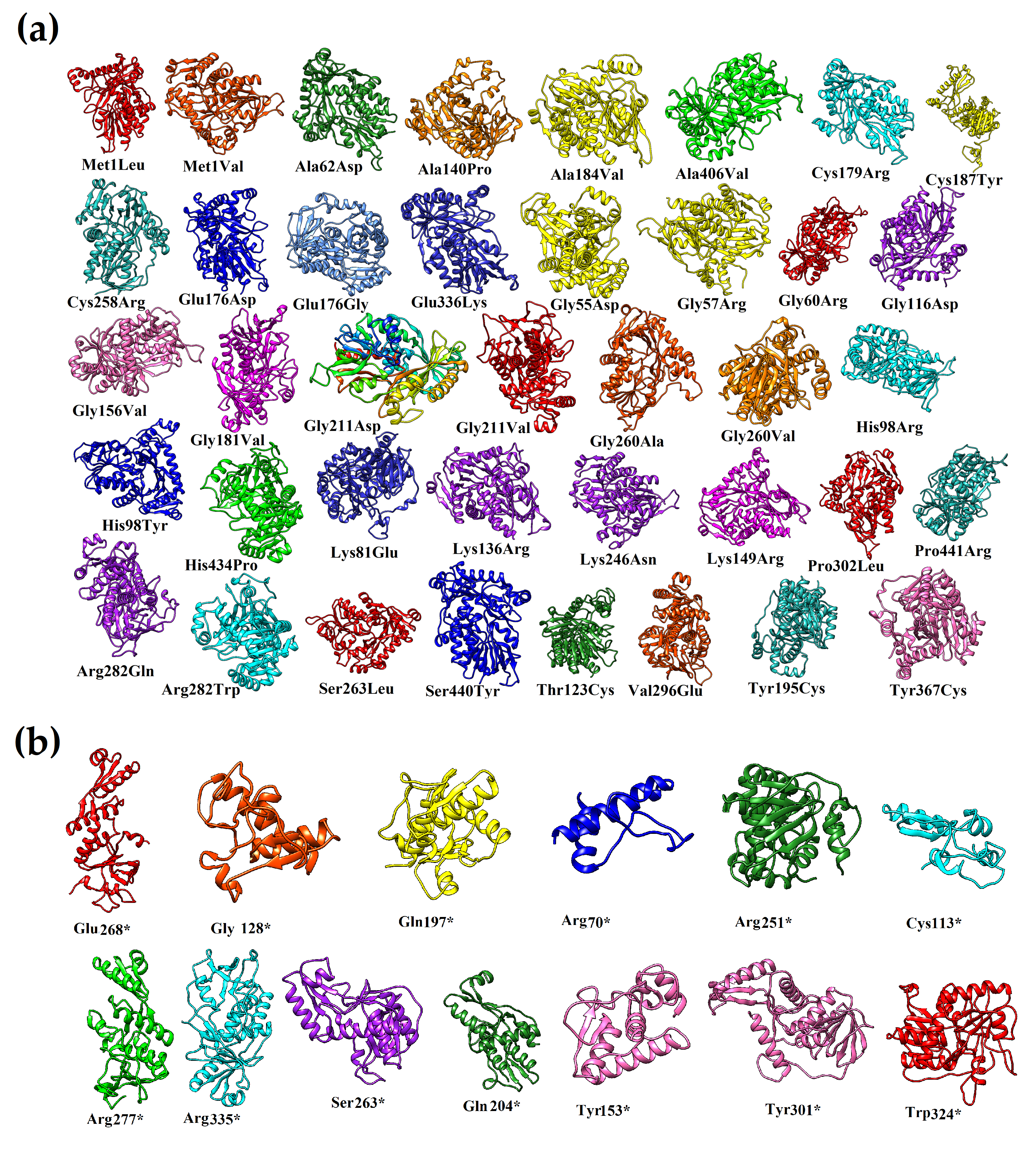

Supplement: Supplementary file 1 [file genes-13-00698-s001.zip › genes-1649920-supplementary/supplementary files/Figure S1.tif]

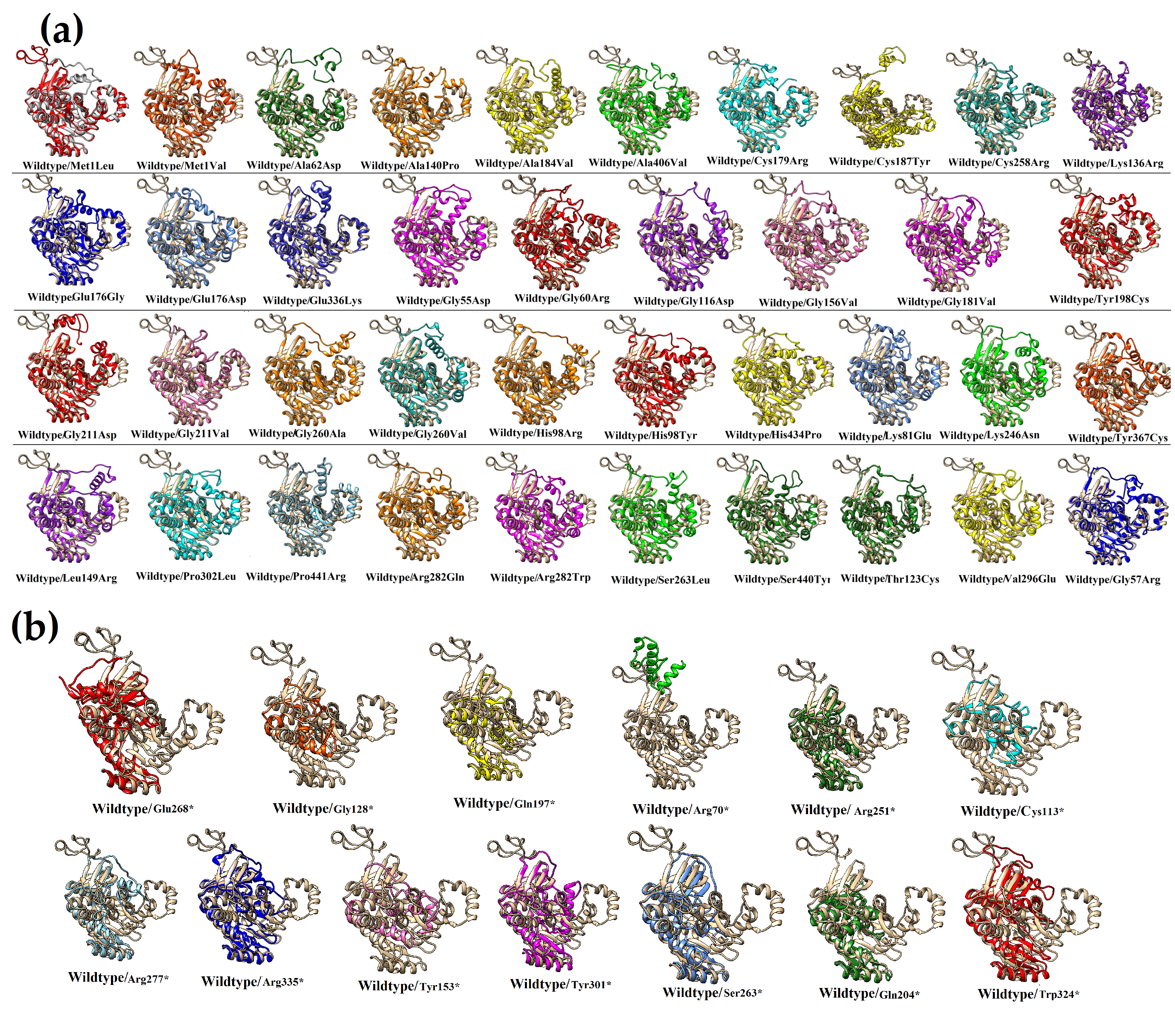

Supplement: Supplementary file 1 [file genes-13-00698-s001.zip › genes-1649920-supplementary/supplementary files/Figure S2.tif]

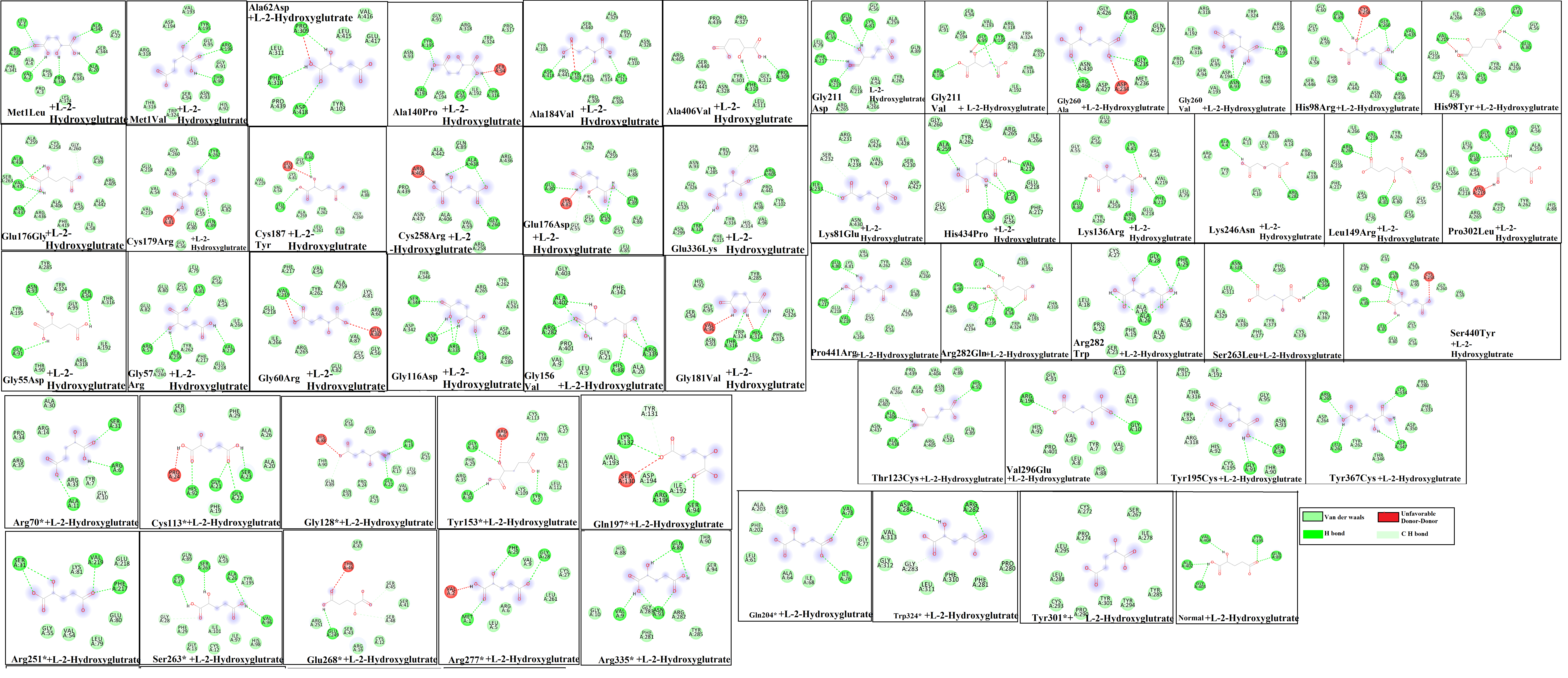

Supplement: Supplementary file 1 [file genes-13-00698-s001.zip › genes-1649920-supplementary/supplementary files/Figure S4.tif]
